# Inside‐out tibial tunnel drilling technique is a reliable approach for all‐inside ACL reconstruction: A longitudinal MRI assessment

**DOI:** 10.1002/jeo2.70068

**Published:** 2024-11-10

**Authors:** João Pedro Oliveira, Otília C. d'Almeida, Ricardo Sampaio, José Carlos Noronha

**Affiliations:** ^1^ Faculty of Medicine University of Coimbra Coimbra Portugal; ^2^ Orthopaedic Department Hospitais da Universidade de Coimbra, Unidade Local de Saúde de Coimbra Coimbra Portugal; ^3^ Coimbra Institute for Biomedical Imaging and Translational Research (CIBIT‐ICNAS) University of Coimbra Coimbra Portugal; ^4^ Department of Radiology Hospital Lusíadas Porto Oporto Portugal; ^5^ Venerável Ordem Terceira de São Francisco Oporto Portugal

**Keywords:** all‐inside technique, anterior cruciate ligament reconstruction, inside‐out tibial tunnel, tibial socket, tunnel widening

## Abstract

**Purpose:**

To longitudinally evaluate sockets localization, tunnel morphological changes and graft maturation after the *inside‐out tibial tunnel drilling technique for all‐inside Anterior Cruciate Ligament Reconstruction (ACLR)*. We hypothesized that due the necessary angle for the inside‐out reaming procedure, the described technique could input changes in the tibial socket.

**Methods:**

Fourteen knees treated with the same all‐inside ACLR technique were randomly assigned for a magnetic resonance evaluation. All patients were operated by the same surgeon and performed the same follow‐up rehabilitation protocol. Socket's localization, shape and widening, as well as graft maturation and integration, were evaluated intraoperatively at 6 months and 4 years after surgery.

**Results:**

Both femoral and tibial tunnels had an expected increase at 6 months follow‐up. The widening was larger in the tibial tunnel (12.6 ± 10.0% vs. 9.1 ± 8.5%), yet this difference was not statistically different. Tibial tunnel was well centred in the tibial plateau and the integration of the graft was higher in the tibial socket. Four years after surgery, there was a general reduction of diameter in both tunnels. The tunnel occlusion rate was 33.3% for tibia and 16.7% for femur.

**Conclusions:**

Overall, our results show that within a 4‐year follow‐up period, the *inside‐out tibial tunnel drilling technique for all‐inside ACLR* represents a safe technique that did not influence the tibial socket position nor tunnel widening or graft maturation in the long term.

**Level of Evidence:**

Level IV.

**Abbreviations**
ACL: anterior cruciate ligament
ACLR: anterior cruciate ligament reconstruction
MRI: magnetic resonance imaging
n.s.: nonsignificant
ROI: regions‐of‐interest
T_0_
: time of surgery
T_1_
: 6 months after surgery
T_2:_
4 years after surgery
TW: tunnel widening
W1: tunnel aperture
W2: tunnel largest section

## INTRODUCTION

In 1995, Morgan et al. [24] first described the all‐inside technique for Anterior Cruciate Ligament reconstruction (ACLR). This technique involves creating sockets in both the femur and tibia (avoiding complete tunnels), introducing the graft into the knee through an arthroscopic portal and using suspensory cortical fixation devices for graft fixation. Several variations were developed in the last years, being this technique evaluated in comparative studies [5, 10, 18, 21, 22].

One of the theoretical benefits of the all‐inside technique is a lower incidence of tunnel widening (TW) [3, 12, 19, 20, 21]. This is a phenomenon that frequently occurs after ACLR, particularly with hamstring tendon grafts, in the first 6 weeks after surgery [13, 27]. Clinical studies have demonstrated that the tendons are integrated into the bone tunnel by 12 weeks [8, 25]. An expected decreasing of the tunnels diameter normally occurs 1–2 years after surgery [5, 19] and, the main clinical concern of TW is ACL late integration, biomechanical graft insufficiency and bone loss in case of ACL revision surgery.

**Figure 1:**
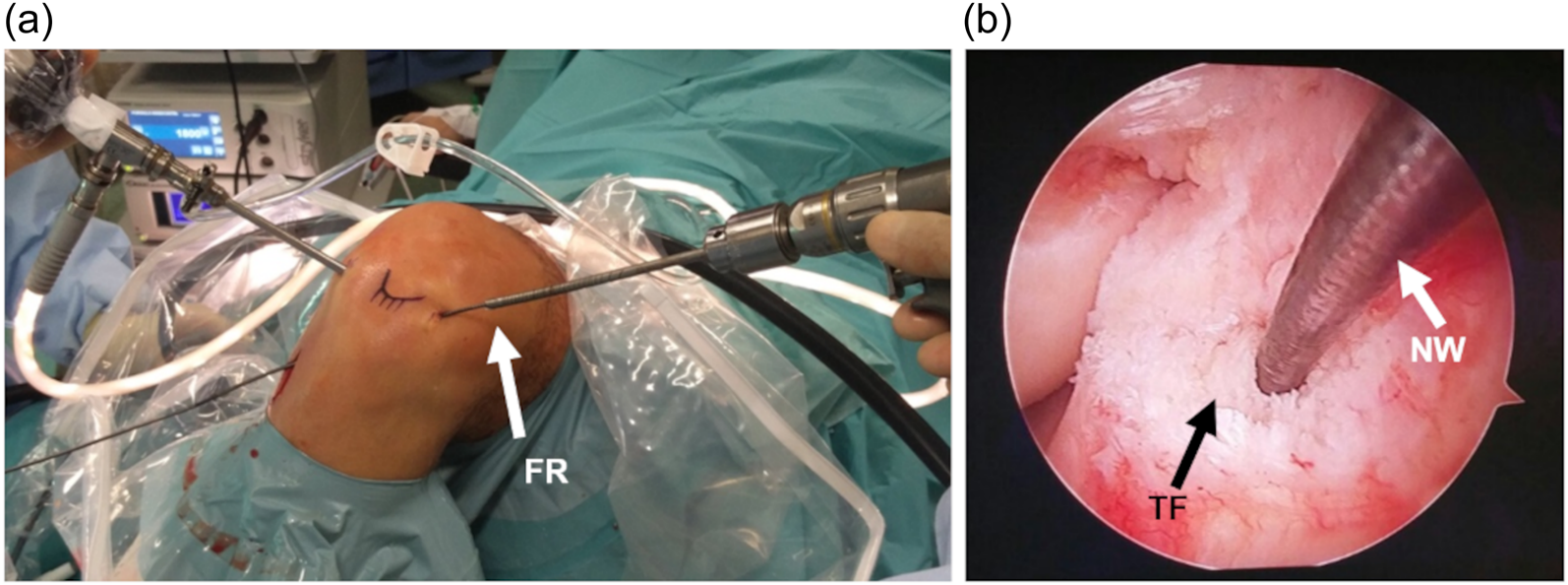
Inside‐out tibial tunnel drilling technique for all‐inside ACLR (left knee). (a) Operation room view; (b) Intra‐articular view. ACLR, anterior cruciate ligament reconstruction; FR, flexible reamer; NW, nitinol wire; TF, tibial ACL footprint.

In 2018, Noronha and Oliveira [23] described an arthroscopic all‐inside ACLR technique where the tibial socket was made with flexible reamers in an inside‐out fashion using a quadrupled semitendinosus autograft (Figure 1). For fixation, both in tibia and femur, cortical adjustable suspensory fixation devices were used.

To our knowledge, there have been no studies evaluating the tibial and femoral morphologic tunnel changes, graft maturation and sockets localization after an *inside‐out tibial tunnel drilling technique for all‐inside ACLR*. We hypothesized that, due to the necessary angle for the inside‐out reaming of the tibial tunnel, it could be nonanatomic and an increased widening could occur with consequent early ACL graft rupture, in comparison to other previously described ACLR techniques.

## MATERIAL AND METHODS

Patients who underwent an *inside‐out tibial tunnel drilling technique for all‐inside ACLR* [22], between December 2017 and July 2019, performed by a single senior surgeon and in the same Institution, were considered for study eligibility. Patients were excluded if they had sustained a multiligament injury, but patients with meniscal and/or chondral injuries were included. Further exclusion criteria included having history of previous knee injury/surgery and body mass index >30 kg/m^2^. Written informed consent was obtained from all the patients. Approval for conducting this study was conceded by the Institutional Review Board Ethical Committee, under the reference CE‐085/2014.

### Surgical technique

A 4‐strand graftlink semitendinosus graft construct and two adjustable cortical suspensory fixation devices (TightRope RT, Arthrex) were used for tibia and femoral fixation. The femoral socket was made through a standard anteromedial portal with the knee at 120° and the tibial socket placement was made using a 55° tibial drill guide aimer. After the passing pin reached the desired tibial location, a 4.5‐mm cannulated drill bit was used to ream the tibial cortex, and posteriorly both the drill bit and pin were removed. At this stage, a flexible wire went through the created hole in the tibia and exit by the anterolateral portal. Finally, in an inside‐out fashion, a canulated flexible reamer progressed through the wire from the anterolateral portal to the tibial plateau, with the same diameter of the graft, creating the tibial socket (Figure 1). A Smith and Nephew (Andover) Clancy anatomic cruciate guide flexible drill system was used and, after pretensioning the graft at 80–90 N for 15 min and cycling the knee about 20 times, final fixation was done at full extension.

### Postoperative rehabilitation

All patients were placed in a partial extension brace for 4 weeks, only during sleeping time. For the first 4 weeks, partial weight bearing was allowed and isometric exercises were initiated on the day after. Next, patients progressively started to participate in functional activities, including running at 4 months, and a return‐to‐sports‐specific training was allowed between 7th and 9th month after surgery, depending on functional and clinical outcomes.

### Tunnels evaluation

Drill diameter at time of surgery (T_0_) was registered, for both tibia and femoral sockets, during ACLR. At two distinct follow‐up periods (6 months [T_1_] and 4 years [T_2_]) tunnel diameter, sockets localization and graft maturation were also assessed by magnetic resonance imaging (MRI). Eventual complications, such as ganglion cysts in the tunnels, were also recorded.

All MRI studies were conducted in 1.5 T scanners. Measurements were digitally performed on standard proton density (repetition time: 3000–3500 ms, time to echo: 10–30 ms) images with fat saturation (PD‐FatSat) in the sagittal and coronal planes using an OsiriX MD 11.0 workstation (Pixmeo).

**Figure 2:**
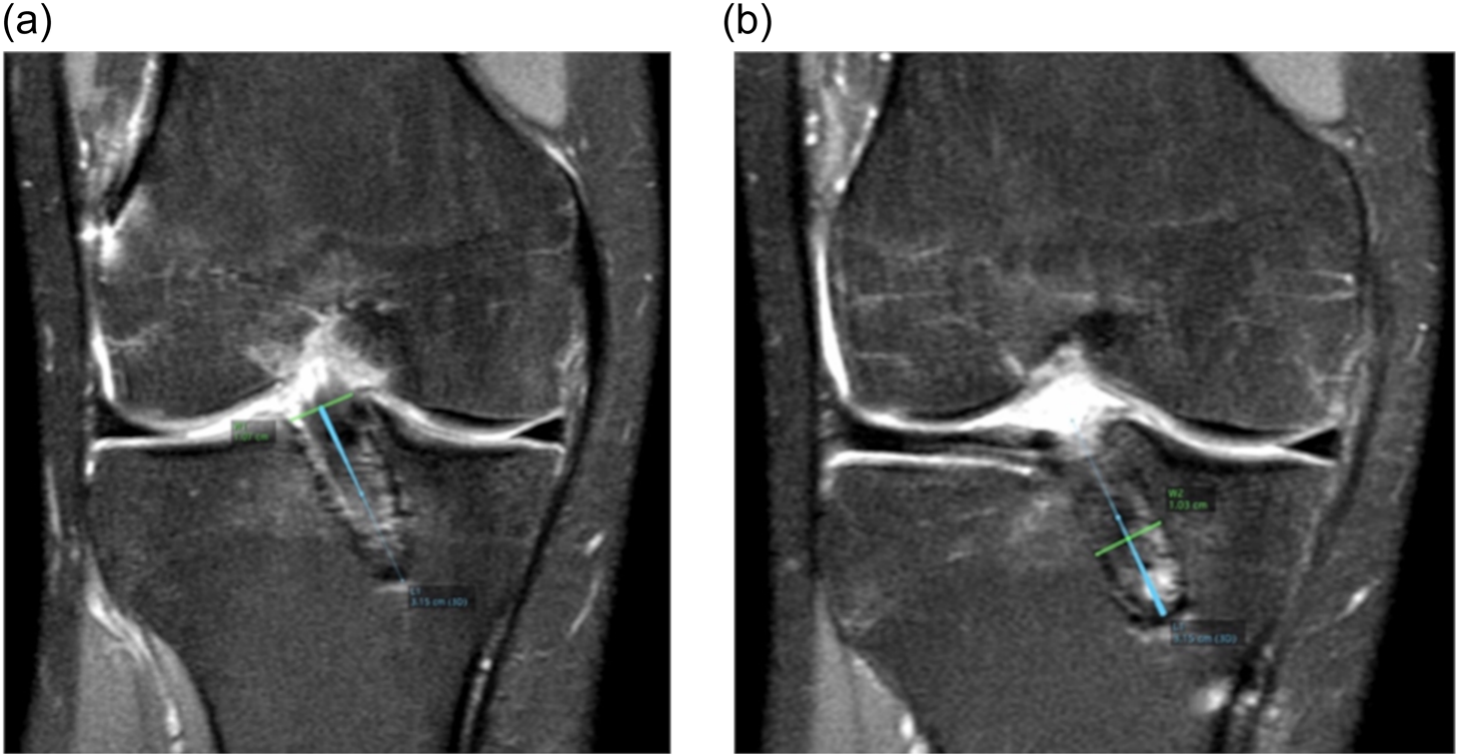
Coronal PD‐FatSat images (right knee). (a) Tibial tunnel diameter at the origin of the tunnel; (b) At the largest section of the tunnel.

Two measurements for tunnel diameter were estimated in both tibial and femoral tunnels, one at the aperture of the tunnel at the intercondylar notch (W1) and other at the largest section of the tunnel (W2) (Figure 2).

**Figure 3:**
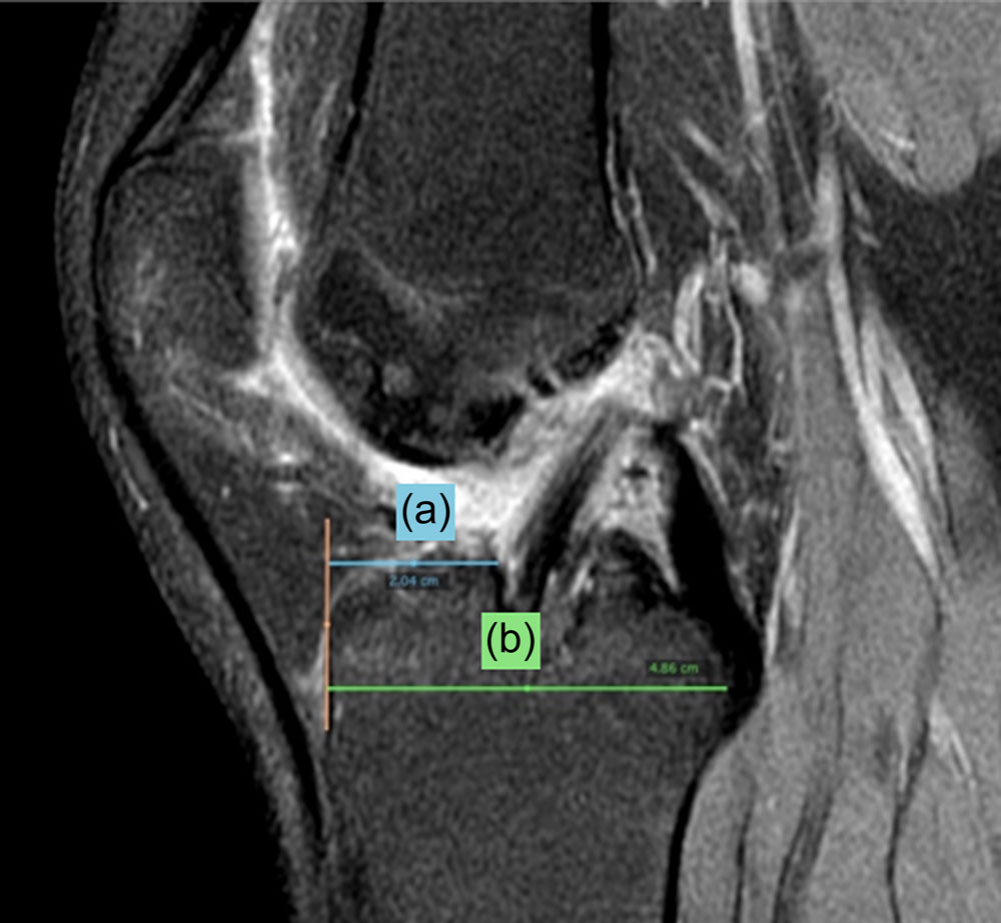
Position of the tibial tunnel, assessed by the Amis and Jacob method, in sagittal PD‐FatSat image. (a) Distance from the anterior border of the tibia to the anterior margin of the tibial tunnel aperture; (b) Anteroposterior diameter of the tibial epiphysis.

**Figure 4:**
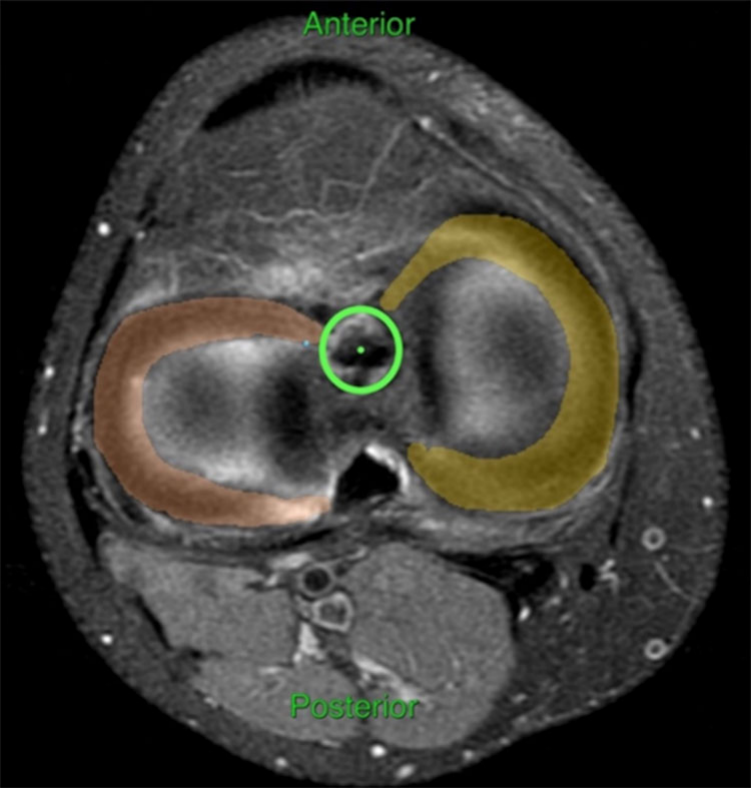
Axial PD‐FatSat image. Green circle—tibial tunnel aperture. For reference, the menisci are coloured.

The position of the tibial tunnel in the sagittal plane was assessed by the Amis and Jacob method [1] in sagittal PD‐FatSat images (Figure 3). In the coronal plane, the tibial tunnel aperture was classified as normal if at the tibial ACL footprint and medially (−) or laterally (+) if off the footprint (Figure 4). The position of the femoral tunnel aperture in the femoral ACL footprint was assessed by a modified Bernhard and Hertel quadrant method [2]. Four distances were measured on the digital printout of the profile view, in millimetres.

**Figure 5:**
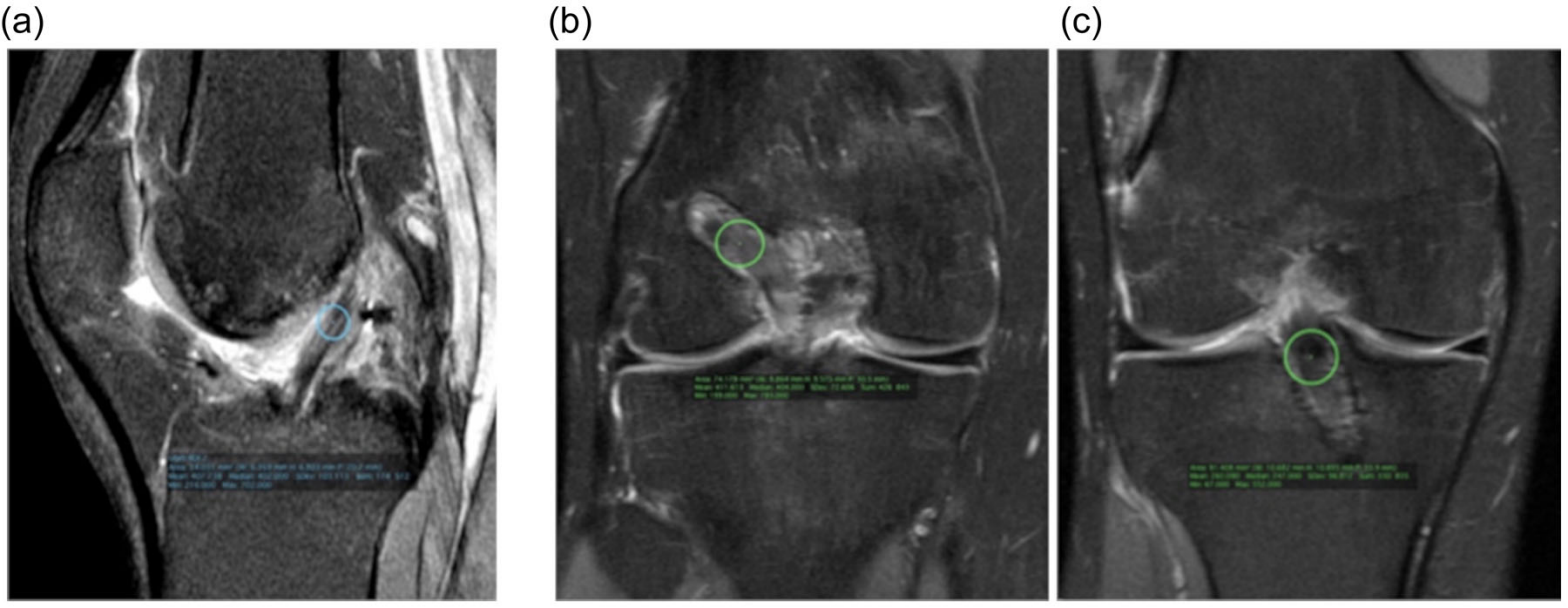
Evaluation of graft maturation in PD‐FatSat images. Regions‐of‐interest (ROI) were drawn. The result is an indirect indication of the collagen content of the graft. (a) Evaluation of graft maturation, sagittal view. (b and c) Evaluation of graft integration in the tunnels, coronal view: (b) Femoral tunnel; (c) Tibial tunnel.

The graft maturation was semi‐quantitatively assessed inside the femoral and tibial tunnels and in the intraarticular portion of the graft. For the intraarticular portion of the graft, two regions‐of‐interest (ROI) were drawn on the graft, and the average signal intensity derived from those two measurements was divided by the signal intensity of the patellar tendon. The result ([Graft ROI_1 _+ Graft ROI_2_]/ROI patellar tendon) is an indirect indication of the collagen content of the graft. For the intra‐tunnel stumps of the graft, an ROI was drawn inside each tunnel from wall to wall in the proximal third of the tunnel, and the signal intensity in this ROI was divided by the signal intensity in the ROI placed in the patellar tendon in the same MRI sequence (Figure 5).

Tibial tunnel inclination was evaluated at the coronal plane by the angle between the horizontal plane defined by the tibial plateau and the centreline of the tibial tunnel.

An additional rater measured W1 at 6 months postoperative (T_1_) for eight cases. Interrater reliability on W1 MRI measurements at T_1_ was good for both tibia (intraclass correlation coefficient [ICC] = 0.911) and femur (ICC = 0.766).

### Statistical analysis

All statistical analyses were carried on IBM SPSS Statistics for Windows (version 28, IBM Corp.).

Qualitative data were expressed in absolute or relative frequencies, and quantitative data with mean ± SD or median (IQR), as appropriate. One‐sample *t* tests were applied to evaluate the difference of W1 between surgery (drill diameter) and the first MRI assessment, and two samples *t* test to compare TW between tibia and femur after verifying data normality with the Shapiro–Wilk test. Reliability of the estimation of W1 was evaluated for tibia and femur tunnels at 6 months. ICC average measures were calculated based on a mean rating (k = 2), absolute agreement, two‐way mixed‐effects model. Statistical tests were performed bilaterally for a significance level set at 5%.

The sample size was calculated using G‐Power 3.1, for two‐sided hypothesis one sample *t* tests at significance level of 0.05 and a target power of 80%. Effect sizes were considered with data reported by Eichinger et al. and Mayr et al. [5, 19]. Assuming an effect size of 1 for comparing changes in tibial TW between baseline and 6 months, a minimum of 10 knees was required. As a larger diameter difference would be expected between 6 months and 4 years, considering an effect size = 1.5, the estimated minimum sample size required was six knees.

## RESULTS

A series of 14 knees (two patients had bilateral surgery) submitted to an *inside‐out tibial tunnel drilling technique for all‐inside ACLR* was retrospectively included in this study. The mean age at surgery was 27.2 ± 12.1 years old (age range: 13–50 years). Our cohort included 11 male and one female patient (two bilateral cases within male patients). There were five patients with concomitant meniscal lesions. All participants were submitted to MRI within 4.9–6.7 months postsurgery (mean, 5.8 ± 0.7 months follow‐up) and seven of them repeated the MRI within 42.2–60.5 months postsurgery (mean, 48.8 ± 7.2 months follow‐up), but in one case, the MRI exam was corrupted. In all the 14 ACLR procedures, ACL graft did not show signs of re‐tearing.

### TW

**Figure 6:**
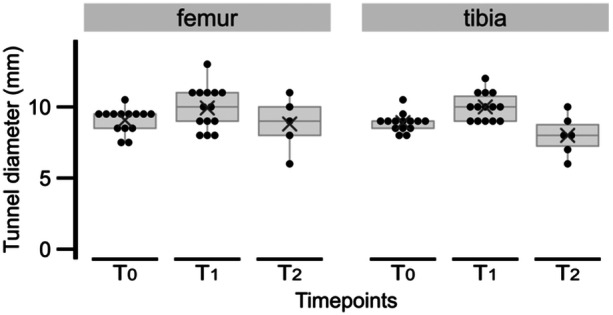
Diameter of the femoral and tibial tunnels at the intercondylar notch (mm) at surgery (T_0_, *n* = 14 knees), 6 months follow‐up (T_1_, *n* = 14 knees) and 4 years follow‐up (T_2_, *n* = 6 knees). Boxplots are overlaid by the individual values and the × depicts the mean. The figure was produced using the ggplot2 package in R version 4.2.1.

Tunnel diameter at time of surgery (T_0_) and at two follow‐up periods (T_1_, T_2_), for both tibia and femoral sockets, was registered (Figure 6).

Both tibial (absolute change: t_13_ = 5.0, *p* < 0.001; %Change: t_13_ = 4.7, *p* < 0.001) and femoral (absolute change: t_13_ = 3.8, *p* = 0.002; %Change: t_13_ = 4.0, *p* = 0.001) articular diameter (W1) had a significant increase after 6 months follow‐up. The widening was larger in the tibial tunnel (1.1 ± 0.8 mm [12.6 ± 10.0%] vs. 0.9 ± 0.8 mm [9.1 ± 8.5%]), yet this difference was not statistically different between the tibial and femoral W1 widening (absolute change: t_13_ = 1.0, *p* = 0.355; %Change: t_13_ = 1.2, *p* = 0.257).

**Figure 7:**
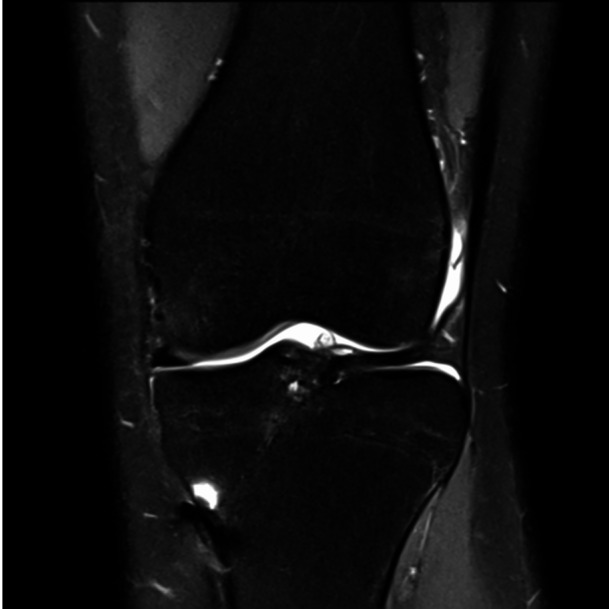
Coronal PD‐FatSat image showing tibial tunnel fully occluded with bone at 4 years follow‐up.

From the six knees re‐assessed after 4 years, there was occlusion in two tibial tunnels (Figure 7), in which one became also fully occluded at both sections of the femoral tunnel. From 6 months to 4 years postsurgery, tibial W1 has not enlarged in any of the evaluated cases, and in fact in 83.3% (5/6), it decreased (mean diameter, 10.0 ± 1.0 vs. 8.0 ± 1.4). In this period, femoral tunnel diameter at W1 has not enlarged in 83.3% (femoral W1 decreased in three cases, in which one became obliterated; two cases maintained its diameter; mean diameter, 9.9 ± 1.5 vs. 8.8 ± 1.9). Radiological evaluation is shown in Table 1.

**Table 1:** Radiological characterization of the femoral and tibial tunnels at follow‐up (6 months and 4 years).

Radiological characterization		6 months (*n* = 14)	4 years (*n* = 6)
Femoral tunnel outcomes			
	Articular diameter (W1), mm	9.9 ± 1.5	8.8 ± 1.9^a^
	Middle diameter (W2), mm	9.6 ± 1.7	7.8 ± 2.2^a^
	Tunnel shape, cylindrical:fusiform, *n* (%)	10:4 (71.4: 28.6)	1:4 (20.0: 80.0)
	Modified Bernhard‐Hertel (Blumensaat line), %	33.3 ± 4.6	‐
	Modified Bernhard‐Hertel (Intercondylar notch height), %	33.4 ± 6.4	‐
	Tunnel occlusion rate, *n* (%)	0.0	1 (16.7)
Tibial tunnel outcomes			
	Articular diameter (W1), mm	10.0 ± 1.0	8.0 ± 1.4
	Middle diameter (W2), mm	9.9 ± 1.1	7.8 ± 1.0^b^
	Coronal inclination of the tunnel (deg)	59.9 ± 4.1	‐
	Tunnel shape, cylindrical:fusiform, *n* (%)	6:8 (42.9: 57.1)	3:1 (75.0: 25.0)
	Amis‐Jacob ratio, %	40.3 ± 2.8	‐
	Coronal footprint position (*N*: −: +)^c^	13:0:1	‐
	Tunnel occlusion rate, *n* (%)	0.0	2 (33.3)

^a^ One case of femoral tunnel occlusion in both sections, W1 and W2.

^b^ Two cases of tibial tunnel occlusion in W2 section.

^c^ N, normal; −, medial deviation; +, lateral deviation.

### Tunnel location

Tunnel locations are presented in Table 1. The Amis–Jacob ratio used for evaluation of the tibial tunnel was centred 40.3 ± 2.8% posteriorly in the tibial plateau from which 92.9% of the cases were well centred on the coronal plane (one case was laterally deviated). The centre of the femoral tunnel was 33.4 ± 6.4% of the height of the intercondylar notch and 33.3 ± 4.6% along the Blumensaat's line. The inclination of the tibial tunnel was assessed at the coronal view and the mean value was 59.9 ± 4.1°.

### Graft maturation

Graft maturation was semi‐quantitatively assessed inside the femoral and tibial tunnels and in the intraarticular portion of the graft at 6 months follow‐up. The tunnels were hyperintense at all locations, being the intensity of the signal, in general, higher in the femur (6.4 ± 2.0) than in the intraarticular (5.1 ± 1.9) or tibial (4.0 ± 1.8) portion of the graft.

## DISCUSSION

In this longitudinal study, we found an expected increase of the tibial and femoral tunnels 6 months after all‐inside ACLR [7, 9, 16, 19, 21] with the *inside‐out tibial tunnel drilling technique*. Additionally, between mid‐term and the 4th year of follow‐up, most tunnels become smaller, in agreement with studies reporting a decrease after the first year [5, 19]. Moreover, tunnel occlusion rates of 33.3% and 16.7% were achieved for tibia and femur, respectively.

According to Amis and Jakob [1], who described a method to identify the anterior–posterior tunnel position in the tibia, in percentage terms, the anatomical ACL attachment was located between the tip of the lateral tibial spine and the small ‘third spine’ on the edge of the medial tibial plateau, being 43% posterior on the tibia. More recently, Neil et al. [15] conducted an MRI study and found that the position of the ACL insertion into the tibia as a percentage of the anterior‐posterior and the mediolateral width was 37.2 ± 5.5% and 47.4 ± 1.5%, respectively. Our analysis showed that the tibial tunnel was placed 40.3 ± 2.7% posteriorly in the tibial plateau and, in 92.9% of the cases, well centred medio‐laterally, indicating that the *inside‐out tibial tunnel drilling technique* will not change the tibial socket placement.

Simmons et al. showed that a tibial tunnel angled at 60°–65° to the tibial articular surface on the coronal plane would allow an ACLR that would reproduce the graft tension pattern of the native ACL [30]. This data was recently confirmed in the MRI study by Neil et al. where the ACL angle with the tibial plateau in the coronal plane was 69 ± 5.5° [15]. In our study, the coronal inclination of the tibial tunnel was 59.9 ± 4.1°, tending to be less vertical as the published data [15, 28].

With the all‐inside ACLR techniques, graft fixation can be achieved using cortical button devices on the femoral and tibial sides and sockets that are created instead of complete tunnels, preserving bone stock and maintaining the cortical bone in an intact status. The graft has full contact within the bone socket without any foreign material, which may allow early graft integration [31] and decrease the risk of synovial fluid leakage as well, as it prevents the allergic reactions and biologic or immune responses with the use of biodegradable screws [14] allowing, at the same time, a 360° contact between the bone and the graft.

Moreover, the pathophysiology of TW is multifactorial. Mechanical, surgical and biological factors have all been implicated in the aetiology [4, 6, 17, 28]. Mechanical factors reported to affect TW include malposition of the graft, aggressive rehabilitation and increased graft forces due to improper graft placement [10, 33]. Biological factors include the surface area for tendon–bone healing, influx of synovial fluid into the tunnel, nonspecific inflammatory responses, cell necrosis in the graft during remodelling, immune response, cell necrosis due to drilling and foreign body reactions [10, 12, 27]. Therefore, it was essential to understand that the *inside‐out tibial tunnel drilling technique for all‐inside ACLR* did not change the tibial socket placement, supported by the consistent trend for tunnel diameter decreasing at 4 years of follow‐up.

Tunnel diameter is also an important factor for planning revision cases of ACLR failure. Staged revision needs to be considered if the tunnel diameter is >10 mm, and it may be indicated with diameters larger than 12 mm [26]. In this longitudinal study, the maximum tibial and femur diameter at 6 months was 12 and 13 mm, respectively (one case) and, at the 4 years follow‐up, in none of the evaluated cases of this all‐inside ACLR technique, the benchmark value equal or superior to 12 mm for the tunnel diameter was verified.

In an all‐inside ACLR technique, a button for graft fixation must be used, which allows graft micromovements at the bone–tendon interface (bungee, windshield wiper effects). This can be a concern and partially justify some of the widening of the tunnels, which could be partially controlled if a hybrid ACL fixation is used (cortical fixed‐loop device in the femur and adjustable‐loop device in the tibia), since cumulative peak cyclic displacement of the fixed‐loop devices are significantly lower than the adjustable‐loop [11].

In the beginning of the healing process, the tissue between the graft and the tendon has high signal intensity in PD‐FatSat (highly cellular), close to fluid and synovial membrane, respectively [29]. According to Weiler et al. [32], the return of signal intensity at 24–104 weeks may indicate a certain amount of structural graft restitution. In our study, there was a tendency for the integration of the graft beginning and, at the same time, being higher in the tibial tunnel, followed by the intra‐articular part of the graft, and finally, at the femoral tunnel, at 6 months postoperative.

A limitation of this study includes the loss of follow‐up data on the second time point. However, not only results obtained in the first follow‐up are comparable to other ACLR techniques but also the findings on the second time‐point show a consistent trend. Additionally, the same method was applied to assess morphologically the ACL tunnels between 6 months and 4 years of follow‐up with the same all‐inside ACLR technique.

## CONCLUSION

*Inside‐out tibial tunnel drilling technique for all‐inside ACLR* does not influence the location of the tibial socket nor TW or graft maturation after a follow‐up period of 4 years. Both tunnels had an expected increase at 6 months followed by a consistent decrease verified at 4 years of follow‐up.

## AUTHOR CONTRIBUTIONS

João Pedro Oliveira contributed to the conception and design of the study, participated in data collection and interpretation, wrote the manuscript and prepared the original draft. Otília C. d'Almeida performed the statistical analysis, involved in interpretation of data and contributed to the critical revision of the manuscript. Ricardo Sampaio participated in the collection, analysis and interpretation of MRI data. José Carlos Noronha contributed to the conception and design of the study, performed the surgeries and provided key insights on surgical techniques. All authors read and approved the final manuscript.

## CONFLICT OF INTEREST STATEMENT

The authors declare no conflict of interest.

## ETHICS STATEMENT

All procedures performed in the study involving human participants were in accordance with the ethical standards of the institutional research committee and the 1964 Helsinki Declaration and its later amendments or comparable ethical standards. Ethical approval was conceded by the Institutional Review Board of the Ethical Committee of the Faculty of Medicine of Coimbra University, under the reference CE‐085/2014.

## Data Availability

All data are available from the corresponding author on reasonable request.
